# Breaking the Cycle: Can Vitamin D Bridge the Gap Between Gut Microbiota and Immune Dynamics in Multiple Sclerosis?

**DOI:** 10.3390/ijms26125464

**Published:** 2025-06-07

**Authors:** Feray Gençer Bingöl, Emine Kocyigit, Elif Çelik, Duygu Ağagündüz, Ferenc Budán

**Affiliations:** 1 Department of Nutrition and Dietetics, Faculty of Health Sciences, Burdur Mehmet Akif Ersoy University, Burdur 15100, Türkiye; fgencer@mehmetakif.edu.tr; 2 Department of Nutrition and Dietetics, Faculty of Health Sciences, Ordu University, Ordu 52200, Türkiye; kocyigitem@gmail.com; 3 Department of Nutrition and Dietetics, Faculty of Health Sciences, Süleyman Demirel University, Isparta 32260, Türkiye; elifcelik@sdu.edu.tr; 4 Department of Nutrition and Dietetics, Faculty of Health Sciences, Gazi University, Emek, Ankara 06490, Türkiye; 5 Institute of Physiology, Medical School, University of Pécs, H-7624 Pécs, Hungary

**Keywords:** dysbiosis, immunity, gut microbiota, multiple sclerosis, vitamin D

## Abstract

Multiple Sclerosis (MS) is a chronic disease with autoimmune and neurodegenerative features that affect the nervous system. Genetic predisposition and environmental factors, such as vitamin D deficiency and dysbiosis activating a pro-inflammatory response, have a role in the etiology of the disease. In this context, the interactions of vitamin D with the gut microbiota and immune system have attracted attention in recent years. Vitamin D (1,25-dihydroxycholecalciferol) modulates the immune response by binding to the Vitamin D receptor (VDR). This pathway supports the functions of regulatory T cells by suppressing the activity of T helper cells 1 and 17 (Th1 and Th17). In MS patients, dysbiosis is characterized by a decrease in microbial diversity, and an increase in pro-inflammatory species is observed when compared to healthy individuals. Vitamin D has protective effects on eubiosis via VDR in intestinal epithelial cells, also reducing intestinal permeability by regulating tight junction proteins. In this way, vitamin D may contribute to the prevention of systemic inflammation. Although the relationship between vitamin D and the immune system is well documented, studies that address the triad of vitamin D level, gut microbiota, and immune response in MS are still limited.

## 1. Introduction

Multiple sclerosis (MS) is a chronic autoimmune illness mediated by the immune system that causes neurodegeneration in the central nervous system (CNS), resulting in demyelination as a result of an inflammatory response [1,2,3]. The number of people with MS worldwide has risen from 2.3 million in 2013 to 2.8 million in 2020 and then to 2.9 million in 2023 [4]. According to data from 75 reporting countries, the incidence is 2.1 per 100,000 people/year. It is twice as high in women as in men [5]. In the study by Khan et al. in 2025, when the data from the 2021 Global Burden of Disease Study were evaluated, it was stated that 1.89 million people worldwide were living with MS and more than 62,000 new cases were diagnosed in 2021 [6]. The disease is mainly classified as relapsing–remitting (RRMS), primary progressive (PPMS), or secondary progressive (SPMS), depending on the clinical course [7]. RRMS is characterized by relapses followed by periods of partial or complete recovery. SPMS and PPMS are defined by the progression of disability over time. Traditionally, disease progression was closely related to relapsing activity. However, evidence has highlighted the concept of progression independent of relapse activity (PIRA), which refers to the gradual accumulation of disability in the absence of clinical relapses. PIRA is thought to reflect underlying neurodegenerative processes that are distinct from acute inflammatory demyelination [8]. It has been stated that inflammation and neurodegeneration are not independent of each other but are part of a disease continuum, and that age is a determining factor in disease progression [9].

Numerous factors impact the development of MS [10,11,12,13,14,15,16]. Family history, particularly having a first-degree relative with MS, increases susceptibility. Specific genetic markers, such as the HLA-DRB1*15:01 allele, have been strongly associated with elevated risk [15,17]. Among environmental contributors, vitamin D (1,25-dihydroxycholecalciferol) deficiency has been linked to a higher incidence of MS [12,13]. Additionally, the Epstein–Barr virus infection, particularly when contracted later in life, is a recognized risk factor [10,18,19]. Smoking is linked to a higher risk of developing MS as well as the advancement of the illness [10,20,21]. Sociodemographic factors also influence MS risk, with women being affected 2 to 3 times more often than men, and most diagnoses occurring between the ages of 20 and 40 [10,11,16]. Moreover, obesity during adolescence and early adulthood has been implicated in heightened MS susceptibility [14,22].

Recently, the relationship between gut microbiota and the etiopathology of MS has been investigated [23,24,25]. The gut microbiota is associated with different CNS diseases, such as neurodegenerative, neuro-inflammatory, and psychiatric disorders [26,27,28,29,30,31,32,33,34]. Through metabolic, immunological, neurotransmitter, and endocrine mediators, the gastrointestinal tract and the CNS have a reciprocal link known as the gut–brain axis. Eubiosis is important for the maturation and differentiation of neurons and glial cells, maintenance of the integrity of the blood–brain barrier, and regulation of the immune system and inflammation [35]. For example, short-chain fatty acids (SCFAs) are involved in the regulation of the immune system and inflammatory responses. SCFA may increase T regulatory cells (Tregs) by suppressing Jun amino-terminal kinases 1 and p38 pathway and show an anti-inflammatory effect by suppressing T helper (Th) 17 [36,37,38]. In this context, studies on MS have also revealed that gut microbiota changes may affect the disease’s formation and progression [23,24,25,39]. Hence, dysbiosis in the microbiota of patients is relevant in MS [40]. In MS, serum acetate levels, one of the SCFAs, have decreased, while fecal lipocalin-2 levels, which are associated with intestinal inflammation, are increased. RRMS patients have lower level relative abundances of *Alistipes shahii*, *Alistipes finegoldii*, *Anaerobutyricum (Eubacterium) hallii*, *Bifidobacterium adolescentis*, *Coprococcus catus*, *Blautia massiliensis*, *Ruminococcaceae NA sp 34859*, and *Ruminococcaceae NA sp 35056*, and higher level of *Blautia brookingsii* when compared to healthy controls [41]. In another study, the MS group showed a decrease in *Faecalibacterium prausnitzii* and an increase in *Prevotella stercorea* when compared to the healthy control [42]. In general, changes in gut bacterial levels and intestinal bacterial metabolites (SCFA, tryptophan metabolites, etc.) have been shown to be altered in MS [43,44]. The relationship between MS and gut microbiota is two-fold. Gut dysbiosis and increased intestinal permeability create an inflammatory state. In addition, in the case of systemic inflammation, the gut may be affected by impaired communication between the CNS and the enteric nervous system via the vagus nerve [45]. Gut microbiota also affects the permeability of the blood–brain barrier [46,47]. Indeed, the development and course of MS may be influenced by impaired blood–brain barrier permeability [48,49].

Genetic factors [50,51,52,53], infections [54,55], pollution [56,57], stress [58,59], and lifestyle factors such as eating habits [50,60,61,62], which are associated with MS pathogenesis, have also been shown to affect the gut microbiota. However, the interactions between vitamin D, the gut microbiota, and the immune system may be one of the determining mechanisms in the pathogenesis of MS. Vitamin D receptor (VDR) and microbiota composition are correlated. Vitamin D and the VDR may influence the composition and function of the gut microbiota, promoting bacterial diversity and mitigating inflammatory processes by enhancing intestinal barrier function. Meanwhile, metabolites derived from microbiota may modulate immune cell activity, supporting vitamin D-mediated anti-inflammatory responses [63,64,65,66]. This is underpinned by the fact that VDR genetic polymorphism is associated with MS incidence, among others [67]. The purpose of this review is to examine the complex interactions between vitamin D, the immune system, and the gut microbiota in the context of MS, emphasizing their roles in the etiology, development, and possible treatment approaches of the condition.

## 2. Immune Dysfunction and Microbiota Alterations in Multiple Sclerosis

**Figure 1:**
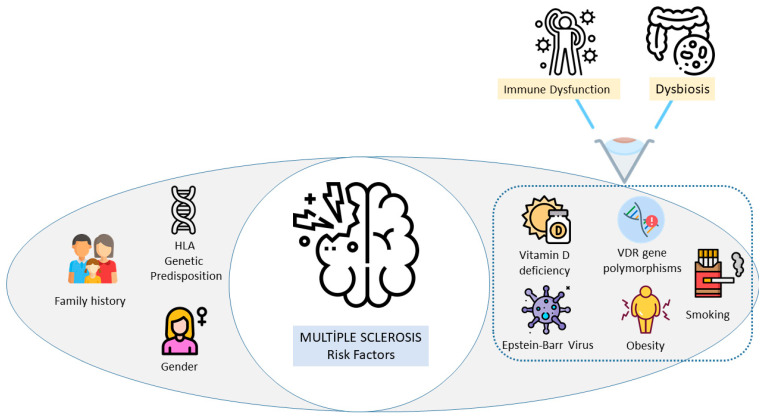
Link between risk factors and MS via immune–microbiota interplay.

Genetic predisposition (such as family history, VDR gene polymorphism, and HLA-DRB1*15:01 allele), environmental factors (including Epstein–Barr virus infection, vitamin D deficiency, and smoking), sociodemographic characteristics (female, 20–40 years), and metabolic/behavioral factors (particularly obesity during adolescence and early adulthood) collectively contribute to the development of MS [10,11,12,13,14,15,16]. A common feature of these environmental and biological factors is the capacity to disrupt immune regulation and gut microbiota composition, thereby facilitating MS pathogenesis. Consequently, the interaction between the immune system and gut microbiota is increasingly recognized as a key mechanistic link through which environmental risk factors (vitamin D deficiency, Epstein–Barr virus infection, smoking, and obesity) exert influence on MS (Figure 1) [68]. This biological framework underscores the necessity of approaching MS not only in terms of individual risk factors but also through a comprehensive understanding of its underlying pathogenic processes.

### 2.1. Overview of the Innate and Adaptive Immune Systems

The immune system comprises two fundamental components: the innate and adaptive immune systems. The innate immune system provides a rapid but non-specific response, aiming to prevent infections through physical, chemical, and cellular barriers. Key elements of this system include the skin, mucosal barriers, phagocytic cells, natural killer cells, and the complement system [69]. The adaptive immune system operates through T and B lymphocytes, offering delayed but antigen-specific and long-lasting protection. T cells regulate cellular immune responses, while B cells produce antibodies that mediate humoral immunity. Additionally, the adaptive immune system generates immunological memory, enabling a faster and more robust response upon re-exposure to the same pathogen. The interaction between these two systems is critical for an effective and coordinated immune response [70,71].

### 2.2. Immune Dysregulation in MS

In the pathogenesis of MS, pro-inflammatory processes and immune system dysregulation, which develop due to the interaction of genetic predisposition and environmental factors, play a crucial role [72]. Myelin sheath injury, inflammation, and CNS lesions are caused by intricate interactions between immune cells, such as T cells, B cells, and myeloid cells, inflammatory signaling pathways, and reactive oxygen species (ROS) production, respectively [73]. Immune tolerance, which normally suppresses the autoimmune response, is impaired in MS, and immune cells enter the CNS and cause tissue damage by secreting inflammatory mediators [74,75].

The breakdown of the blood–brain barrier, activation of local astrocytes and microglia, and finally, neuro-inflammation are all facilitated by high Th cell counts in CNS lesions and cerebrospinal fluid from MS patients. Th1, Th17, Th1-like Th17, Th9, and Th22 are Th cells associated with MS [75]. Th1 cells increase inflammation by secreting interferon-gamma. In contrast, Th17 cells facilitate the entry of immune cells into the CNS by disrupting the permeability of the blood–brain barrier through the production of interleukin (IL)-17 [75,76]. B lymphocytes contribute to antigen presentation, cytokine production, and autoantibody formation. Anti-cluster of differentiation 20 (anti-CD20) immunomodulatory treatments aimed at depleting B lymphocytes, which aim to regulate the immune response, reveal the importance of B lymphocytes in MS [77]. Microglia and macrophages cause myelin destruction through phagocytosis and aggravate inflammation by secreting pro-inflammatory cytokines (e.g., tumor necrosis factor-alpha (TNF-α) and IL-6) [78]. Although Tregs are responsible for suppressing the immune response, the function of these cells is impaired in MS, and the immunosuppressive activity on effector T lymphocytes is reduced. This leads to uncontrolled autoimmune attacks. Treg numbers have been shown to decrease during relapses in MS patients and return to previous levels during remission [79]. Dendritic cells accumulate in MS and play an important role in activating autoimmune T lymphocytes and disrupting immune tolerance [80].

The disruption of tolerance mechanisms triggers immune system dysregulation, and failure to eliminate autoimmune T and B lymphocytes leads to damage in the CNS. Pro-inflammatory cytokines, especially TNF-α and IL-1β, reduce the stability of the blood–brain barrier by increasing permeability [81]. The molecular mimicry mechanism can also trigger the autoimmune process, and the immune system mistakenly attacks the nerve tissue due to the cross-reactivity of some pathogens with myelin proteins [82].

Disruption of the cytokine balance is another important factor in the pathogenesis and progression of the disease. Increased levels of pro-inflammatory cytokines (e.g., IL-1β, IL-6, IL-17, IL-23, IL-12, and TNF-α) and interferon-gamma trigger neuro-inflammation and contribute to the disease progression [83,84,85,86]. However, deficiency or dysfunction of anti-inflammatory cytokines (e.g., IL-4, IL-10, IL-37) or transforming growth factor β is responsible for the chronic and progressive features of MS [86,87].

### 2.3. Dysbiosis in MS: Key Microbial Alterations and Autoimmunity

The gut microbiota maintains immune homeostasis and regulates host immune responses. Dysbiosis, an imbalance in microbiota, has been increasingly associated with autoimmune diseases, including MS. This association suggests that the gut microbiota may potentially drive the disease’s underlying autoimmune processes due to immunomodulatory capacity [88].

A prominent feature of dysbiosis in MS is a reduction in overall microbial diversity, which may potentially lead to a less resilient and functionally impaired ecosystem. In MS patients, various bacterial genera known for immunomodulatory and anti-inflammatory properties are frequently underrepresented. A decrease in SCFA-producing bacteria such as *Firmicutes*, *Roseburia*, *Coprococcus*, *Lachnospiraceae*, *Butyricicoccus*, *Faecalibacterium*, *Dorea*, *Lachnospira*, and *Prevotella* has been reported in MS. Conversely, certain bacterial genera associated with pro-inflammatory responses, including *Bacteroidetes*, *Akkermansia*, and *Ruminococcus* species, are often enriched in MS patients [40].

Altered gut microbiota in MS is thought to contribute to the development of autoimmune responses through various immunological mechanisms. Dysbiosis activates dendritic cells, causing the pro-inflammatory Th1 and Th17 cell differentiation, which play a role in CNS inflammation [89]. At the same time, the decrease in SCFA-producing bacteria negatively affects Treg development and functions, causing impaired immune tolerance and increased autoimmune responses [90]. In addition, imbalances in the intestinal microbiota are associated with the humoral immune response irregularities observed in MS by triggering B cell activity and autoantibody production [91]. The molecular mimicry mechanism also stands out as a potential factor. It is thought that microbial antigens that are structurally similar to myelin proteins may aggravate demyelination processes by activating cross-reactive T cells [92]. Moreover, dysbiosis can compromise the integrity of the intestinal epithelial barrier, leading to increased intestinal permeability. Buscarinu et al. found that increased intestinal permeability was higher in patients with relapsing–remitting MS than in healthy controls. The lactulose/mannitol ratio was significantly higher in the patient group as an indicator of gut permeability. This condition may contribute to leaky gut syndrome, facilitating microbial components and microorganisms into the systemic circulation [93].

### 2.4. Role of Microbiota-Derived Metabolites in Immune Homeostasis

The interaction between the gut microbiota and the immune system is not limited to microbial diversity but also occurs through metabolites produced by microorganisms. Microbial metabolites are bioactive compounds generated through microbial fermentation and metabolic processes. These metabolites significantly regulate the immune system and, consequently, the pathogenesis of MS. In particular, SCFAs, tryptophan metabolites, and bile acid derivatives are among the prominent microbial metabolites in MS pathogenesis [94]. SCFAs are produced by the microbial fermentation of dietary fibers and have regulatory effects on the immune system. SCFAs contribute to immune tolerance by enhancing Treg differentiation and function. Moreover, SCFAs enhance the expression of anti-inflammatory genes by inhibiting histone deacetylases and DNA methyltransferases [95,96,97]. In analyses based on both stool and serum/plasma samples, SCFA levels were generally lower in MS patients compared to healthy individuals [98,99,100]. Decreased levels of SCFAs are negatively associated with pro-inflammatory cytokine levels [101]. On the other hand, some studies have shown that some SCFA types, such as acetate, were increased in MS patients, indicating that SCFAs may have complex and bidirectional effects on immunity [102,103].

**Figure 2:**
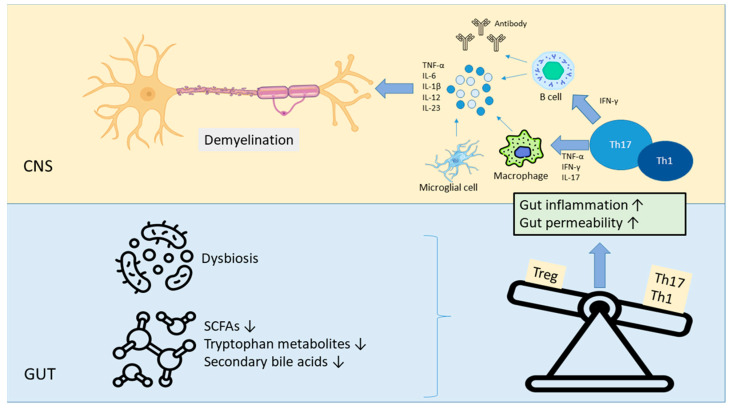
The link between gut microbiota alterations and neuro-inflammation in MS. As a result of dysbiosis, the levels of gut metabolites such as SCFAs, tryptophan metabolites, and secondary bile acids are reduced. This reduction can disrupt the immune balance between Tregs and Th1/Th17 cells, enhancing pro-inflammatory responses. Increased intestinal inflammation and gut permeability may contribute to neuro-inflammatory processes through systemic inflammation. The progression of neuro-inflammation may exacerbate demyelination and negatively affect disease prognosis in MS patients. Down arrow (↓) indicates decrease; up arrow (↑) indicates increase. Abbreviations: CNS, central nervous system; IL, interleukin; TNF-α, tumor necrosis factor alpha; IFN-γ, interferon gamma; Th, T helper; SCFAs, short-chain fatty acids; and Treg, regulatory T cell.

Tryptophan, an essential amino acid, can be metabolized by both the host and gut microbiota into various bioactive compounds, such as indole and derivatives. These metabolites activate the aryl hydrocarbon receptor (AhR), a transcription factor that plays a critical role in regulating the development and function of immune cells. The AhR signaling pathway is essential for maintaining the balance between regulatory Tregs and Th-17 cells, preserving intestinal epithelial integrity and suppressing the production of pro-inflammatory cytokines. Zelante et al. reported that serum levels of the tryptophan metabolite indole-3-carboxaldehyde are inversely correlated with disease duration in MS patients [104]. Secondary bile acids metabolized by the gut microbiota play a crucial role in regulating neuro-inflammation and immune responses in the pathogenesis of MS. In MS patients, a reduction in gut bacteria capable of converting primary to secondary bile acids leads to decreased levels of compounds such as deoxycholic acid and lithocholic acid. This reduction results in the loss of the immunomodulatory effects that promote Treg differentiation and suppress Th17-mediated inflammation. Consequently, the deficiency of these bile acids contributes to immune system imbalance in MS [105]. Both global and targeted metabolomic analyses in adult and pediatric MS populations suggest reduced circulating and fecal bile acid levels [106,107]. Additionally, the presence of bile acid receptors has been detected in MS lesion areas, indicating that bile acids may interact directly with immune and glial cells within the CNS [106]. The role of gut microbiota in MS is described in Table 1. The link between changes in gut microbiota and neuro-inflammation in MS is also summarized in Figure 2. MS is not only a neurological disease but also a multidimensional disease involving immunological and microbial dimensions. Therefore, understanding the interactions between the immune system, microbiota, and microbial metabolites is essential for personalized therapeutic strategies.

## 3. Vitamin D, Gut Microbiota, and Immune Homeostasis: Mechanistic Insights and Clinical Evidence

According to recent studies, gut microbiota is a major environmental risk factor in immune-mediated diseases, such as MS. The gut microbiota may influence MS development and clinical symptoms [110,111]. The gut microbiota significantly influences health and disease, contributing to the human immune system development and activation, preserving against infectious pathogens, helping in food digestion, maintaining energy balance, producing vitamins, and ensuring intestinal barrier integrity [112].

The gut microbiota undergoes continuous changes throughout an individual’s lifespan, influenced by various factors, including dietary habits, pharmacological interventions (notably antibiotics), and psychological stress. The gut microbiota fosters a protective, anti-inflammatory environment that inhibits the growth of pathogenic microorganisms responsible for various illnesses [113,114]. Dietary modifications can alter the bacterial microbiota composition. A Western diet rich in saturated fats and refined sugars yet low in fiber can disturb the balance of gut bacteria and contribute to chronic inflammation. In contrast, fiber, omega-3 fatty acids, and vitamin D_3_ can positively influence microbiota, fostering the growth of microorganisms that produce anti-inflammatory compounds beneficial to overall health [66,115]. Thus, regarding metabolism and gut microbiota, diet can effectively reduce inflammation in both RRMS and primary progressive multiple sclerosis PPMS.

When exposed to sunlight, the skin is the principal site for synthesizing vitamin D, a fat-soluble prohormone [116]. Ergocalciferol (vitamin D_2_), which is derived from plants, and cholecalciferol (vitamin D_3_), which comes from animals, are the two primary dietary forms of vitamin D. Vitamin D appears to play a significant role in the immune system. It influences various processes, including differentiating T cells, activating and growing lymphocytes, and reducing inflammatory cytokines [117,118]. Several studies have linked low serum 25-hydroxyvitamin D (25(OH)D) levels to an increased risk of MS. This vitamin is also involved in the early stages of the disease and impacts its development [119,120]. The occurrence and frequency of MS have been correlated with geographic locations at high latitudes and the duration and intensity of sun exposure. This relationship may be attributed to the reduced levels of ultraviolet radiation in these regions, which subsequently contribute to lower vitamin D levels. Vitamin D plays a crucial role in immune modulation, and insufficient levels of this vitamin, resulting from limited sun exposure, may increase the risk of developing MS by impairing the ability to regulate immune responses and inflammation [120,121].

Vitamin D and VDR signaling are crucial in human health and the disease’s immune-mediated, environmental, genetic, and microbial dimensions [66,122]. Belonging to the nuclear hormone receptor superfamily, VDR controls the expression of genes that regulate vitamin D’s biological impacts. The reaction of 1,25(OH)_2_D_3_ with VDR triggers the activation of transcription [123]. The human VDR is a key gene that significantly influences the regulation of gut microbiota [124]. It has a variety of immunological functions, including suppressing Th17 and Th1 responses, preventing B cell growth and activity, enhancing Treg function, and inducing immune cells to produce antimicrobial peptides [125].

### 3.1. The Effect of Vitamin D on Gut Microbiota Composition

Multiple studies have investigated the relationship between microbiota, their metabolites (such as SCFA), gut homeostasis, and the potential regulatory role of vitamin D/VDR signaling [126,127]. VDRs have demonstrated an impact on the diversity of gut microbes [128]. The fecal microbiota composition is influenced by vitamin D, where higher levels of this vitamin are associated with an increase in beneficial microbial species and a reduction in harmful microbes [129,130]. Given that bacteria are not expressing VDR, the influence of vitamin D levels on gut microbiota is likely an indirect consequence of host response [127].

Vitamin D supplementation does not significantly alter the microbiota diversity of biopsies taken from the sigmoid colon, ascending colon, or terminal ileum; the anticipated predominance of *Firmicutes* and *Bacteroidetes* phyla was seen in healthy people [131]. After 12 weeks of vitamin D treatment in women experiencing vitamin D deficiency, an overall increase in gut microbiota diversity was observed, particularly marked by a rise in *Bacteroidetes* and a decrease in the relative abundance of *Firmicutes*. In addition to improving the *Bacteroidetes* to *Firmicutes* ratio, the results show that *Verrucomicrobia* and *Actinobacteria* phyla levels also rose following vitamin D administration [128]. Further research indicated that vitamin D_3_ administration in individuals with MS increased the prevalence of the mucosal-integrity-promoting species *Akkermansia* in the intestinal tract, together with *Fecalibacterium* and *Coprococcus* [132]. The gut microbiota of young girls showed an increase in *Firmicutes* and *Bifidobacterium* and a decrease in *Bacteroidetes* and *Lactobacillus* after nine weeks of using high-level vitamin D supplements (50,000 IU of cholecalciferol per week) [133]. A comprehensive evaluation of human research (25 studies: 14 interventional and 11 observational) revealed that vitamin D supplementation is strongly associated with alterations in microbiota composition, particularly among the *Actinobacteria*, *Firmicutes*, and *Bacteroidetes* phyla. Concerning alpha and beta diversity, significant vitamin D use seems to induce changes in the composition of bacteria and/or affect the diversity of species [134].

Early studies evaluating microbiota composition in MS patients indicated alterations in specific bacterial genera, including *Akkermansia*, *Prevotella*, and *Methanobrevibacter*. The results presented first suggested that parts of the human gut microbiota may play a role in CNS-specific autoimmunity; however, a consistent pattern did not emerge [135,136]. Axenic mice were colonized with fecal microbiota from the MS patients as well as controls, while accounting for the influence of human genetics on one’s microbiome. The analysis of microbiota composition revealed that the overall microbial profiles exhibited similarities; however, certain bacterial genera, including *Akkermansia*, were found to be elevated in people with untreated MS in contrast to their healthy twins [137]. Limited research has been performed on how vitamin D supplementation affects MS patients’ gut microbiota. The MS patients receiving vitamin D treatment received a 90-day supplementation of vitamin D3 at a dosage of 5000 IU per day. Patients with MS exhibited a rise in the species *Akkermansia*, *Faecalibacterium*, and *Coprococcus* [132].

There is evidence that vitamin D insufficiency alters the microbiota and metabolite composition in the gut. In the mouse model, neither vitamin D treatment nor vitamin D deficiency (in VDR^−/−^ or CYP27B1^−/−^ mice) significantly affected the gut microbiota at the phylum level. However, changes were observed at lower taxonomic levels [138]. In randomized control research with rats, the proportion of *Enterobacteriaceae* increased. Notably, animals without enough vitamin D had higher levels of the related genera *Candidatus Blochmannia*, *Escherichia*, and *Enterobacter*. Rats fed a diet low in vitamin D exhibited a reduction in *Odoribacteraceae*, including the species *Butyricimonas* while showing an increase in *Prevotella* and *Actinomyces* [139]. It also examined the differences between vitamin D receptor knockout mice and CYP27B1 knockout mice and their wild-type counterparts. A systematic review of studies conducted on mice found that the number of *Bacteroidetes* or taxa in that phylum was higher in groups with gene deletions or those on a low vitamin D diet [140].

The effects of vitamin D supplementation can differ based on the dose, duration, and the source of the tissue in microbiota research. These findings suggest that vitamin D is important in influencing gut microbiota diversity. A comprehensive review indicates that vitamin D administration significantly alters the abundance of *Actinobacteria*, *Firmicutes*, and *Bacteroidetes* [66,134]. Metabolic characterization of gut bacteria revealed that metabolic activities associated with fatty acid synthesis and lipid metabolism contribute to the intestinal lumen’s use of vitamin D. Future research, encompassing animal models and randomized controlled trials, should clarify the processes regulating changes in the composition of gut microbiota in relation with vitamin D levels. Furthermore, meticulously structured investigations must provide uniformity in gut microbiota analysis and vitamin D supplementation or evaluation.

### 3.2. The Impact of Gut Microbiota on Vitamin D Regulation

Gut microbiota can significantly influence vitamin D levels and their biological effects, revealing a fascinating interplay that underscores the importance of both nutrition and gut health in overall well-being [141]. Fibroblast growth factor-23 (FGF-23), which is mainly generated by osteocytes and osteoblasts, can directly cause this by inducing CYP24A1 and inhibiting CYP27B1, which prevents vitamin D from being metabolically activated. FGF-23 is an essential vitamin D metabolism regulator, reducing the production of 1,25(OH)_2_D_3_ in proximal renal tubules by suppressing 1α-hydroxylase expression and improving degradation through the upregulation of 24-hydroxylase transcription [142,143]. Germ-free mice exhibited elevated levels of FGF-23, accompanied by hypocalcemia and decreased levels of 1,25-dihydroxyvitamin D and 24,25-dihydroxyvitamin D, in contrast with traditional mice [144].

The gut microbiota may hinder the vitamin’s activity through secondary bile acids, particularly lithocholic acid, that interfere with vitamin D for binding to and stimulating the VDR. The VDR engages with lithocholic acid to enhance CYP24A1 mRNA expression in the ileum, leading to the inactivation of calcitriol [145,146]. Metabolic byproducts of bacteria, particularly SCFA-like butyrate, enhance the intestine expression of VDR via mitigating inflammation [147]. Intestinal vitamin D insufficiency is associated with inflammation, decreased colon length, aberrant mucosal architecture, infiltration of inflammatory cells, and a reduced mucus layer due to changed microbial composition, particularly an increase in *Akkermansia muciniphila* [138]. Certain bacteria, such as *Sebekia benihana* (CYP-sb3a), *Streptomyces griseolus* (CYP105A1), and *Pseudonocardia autotrophica* (Vdh), produce enzymes that facilitate the hydroxylation of steroids, hence hydroxylating and activating vitamin D [148].

### 3.3. Vitamin D, Gut Microbiota, and Immune Homeostasis

The brain and gut microbiota communicate through various channels, encompassing neuronal and immunological signals [91]. This dual interaction, referred to as the gut–brain axis, underscores the potential for intestinal bacteria and their metabolites to affect the pathobiology of MS. Given the connection between MS and aberrant immunological response, it is possible that gut dysbiosis, which can impact immune control, contributes to the development and course of MS [89]. When the gut microbial ecology is disrupted, intestinal permeability increases, allowing toxins and products from bacteria to enter the circulation, which can cause pro-inflammatory cytokines to be produced and aid immune cell infiltration into the CNS. Furthermore, specific gut bacteria have the potential to directly influence immune responses, facilitating the emergence of autoreactive pro-inflammatory T-cell subsets [149].

Vitamin D modulates immune responses that are both innate and adaptive, which is consistent with the expression of VDR in the majority of immune system cells [150]. The VDR is found in macrophages, dendritic cells, activated T cells, and various other cell types, as well as in approximately 30 distinct organs, including the gut [151]. Binding its principal ligand, 1,25(OH)_2_D_3_, primarily activates the VDR, and the VDR facilitates practically all of the physiological actions of vitamin D [152]. The VDR forms a heterodimer with the retinoid-X receptor (RXR) and then binds to vitamin D response elements (VDREs) located in the regulatory regions of target genes. Antimicrobial peptides (AMPs), including cathelicidin, β-defensin, the 25-hydroxyvitamin D 24-hydroxylase (CYP24) gene, and the cytochrome P450 family 11 subfamily A (CYP11A1) gene are part of a wide range of over 1000 genes that contain binding sites for VDRE [153,154,155].

Vitamin D supplementation has been shown to influence immune system modulation in patients with MS. Across various clinical trials, different dosages of cholecalciferol have been administered to assess the effects on cytokine production and immune cell activity, particularly with regard to T cell subsets. The results consistently indicate that vitamin D supplementation can reduce pro-inflammatory cytokines such as IFN-γ and IL-17, suggesting a potential role in controlling inflammation. Additionally, alterations in cytokine ratios, including TNF-α, IL-6, IL-5, and IL-10, have been observed, further supporting the hypothesis that vitamin D exerts regulatory effects on immune responses [156,157,158,159,160].

**Figure 3:**
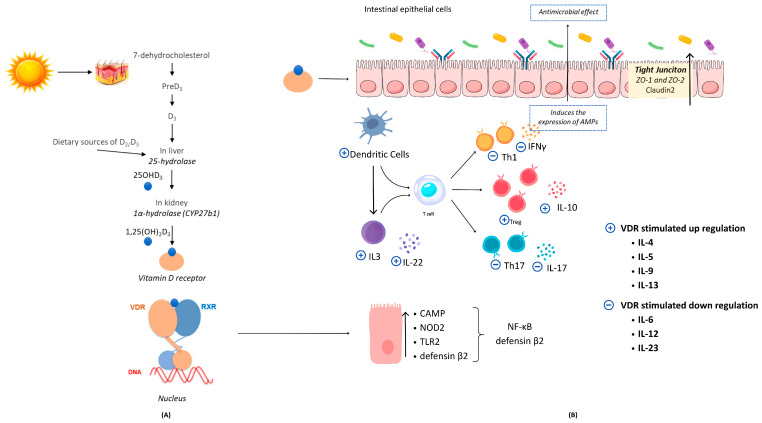
Vitamin D biosynthesis, vitamin D-VDR signaling axis, and the effects of vitamin D on gut microbiota and immunity [64,66,162]. Sunlight exposure or consumption of foods high in vitamin D can produce vitamin D. Bioactive vitamin D acts as the main ligand for vitamin D receptors. The active VDR regulates the expression of 3% of the genome by binding to vitamin D sensitive sites (**A**). Over 1000 genes associated with VDRE have binding sites, including CAMP, NOD2, defensin β2, and TLR2. The activated VDR modulates the expression of intestinal tight junction proteins ZO-1, ZO-2, and claudin 2. The VDR facilitates immunological tolerance in the gut by suppressing the proliferation of Th1 and Th17 cells, which are responsible for the production of IL-17 and IFN-γ, while also enhancing dendritic cell-mediated IL-10 production, hence increasing regulatory T cell generation and promoting a Th cell type 2 response (**B**). Abbreviations: AMPs, antimicrobial peptides; CAMP, cathelicidin antimicrobial peptide; IFNγ; interferon gamma; IL, interleukin; NOD2, nucleotide-binding oligomerization domain protein 2; NF-κB, nuclear factor kappa B; RXR, retinoid X receptor; Th, T helper; Treg, regulatory T cell; TLR, toll-like receptor; VDR, vitamin D receptor; and ZO, zonula occluden.

Significant evidence suggests that vitamin D improves phagocytic activity, stimulates the secretion of antimicrobial peptides (including cathelicidin and β-defensin), and elevates the expression of E-cadherin, zonula occludens (ZO)-1, claudin-1, and occludin proteins [122,143,151]. Vitamin D promotes Th2 cell proliferation, thereby enhancing the production of anti-inflammatory cytokines while inhibiting the Th17 and Th1 clones responsible for producing pro-inflammatory cytokines. Additionally, vitamin D promotes the activity of tolerogenic dendritic cells and modulates the expression of angiogenin-4, which exhibits antibacterial properties. The immune cell population with the highest level of VDR expression is CD8pos T lymphocytes. The gastrointestinal tract, particularly the small intestine, contains most of these cells in the body and serves as a primary line of defense against foodborne antigens [64,161]. Because of its multiple functions, low vitamin D levels can interfere with proper barrier function and intestinal homeostasis. Vitamin D impacts the structure of tight junctions, modifies bacterial colonization, and exhibits anti-inflammatory properties via its connection with VDR. Figure 3 illustrates the process of vitamin D biosynthesis, the vitamin D-VDR signaling pathway, and the impact of vitamin D on gut microbiota and immune function [64,66,162].

#### 3.3.1. Vitamin D/VDR-RXR Complex Stimulates the Transcription of Antimicrobial Peptides

In several cell forms, including colon cells, the vitamin D-VDR-retinoid X receptor complex promotes the transcription of AMPs and improves metabolic and phagocytic activities [150]. AMPs, including β-defensin 2/human β-defensin-2 (DEFB4/HBD2) and cathelicidin [human cationic antimicrobial protein (hCAP18)], act as chemotactic agents for inflammatory immune cells and have antibacterial qualities [163,164]. Vitamin D enhances the expression of AMP mRNA and proteins, which include cathelicidin, defensins, and lysozyme in vitro, as well as Ang4 in vivo [165,166]. AMPs, mainly produced by Paneth cells in the gut, play an essential role in shaping microbiota composition. In vivo studies have illustrated this by showing increased translocation of bacteria after the dissection of Paneth cells and heightened vulnerability to colitis or pathogen infections [167]. Activation of TLRs in human macrophages enhances the synthesis of the AMP cathelicidin through a vitamin D-dependent pathway [168]. An intracellular mechanism related with TLR2/1, as described by Krutzik et al. [169], allows human monocytes to synthesize AMPs. This system is vitamin D dependent. Their findings indicate that the amounts of VDR and 1α-hydroxylase expression influence the effect of TLR1/2 activation. When Gram-negative bacteria activate TLR4 by LPS stimulation, vitamin D starts AMP synthesis [64].

Bioactive 1,25(OH)_2_D_3_ increases antimicrobial activity against microbes by downregulating cathelicidin leucine-leucine-37 and upregulating phagosome growth, which in turn causes cathelicidin overexpression [170]. Human monocytic and epithelial cells primarily activate the intracellular pattern recognition receptor nucleotide-binding oligomerization domain protein two to do this. According to the results, AMP activity significantly affects the variety of gut microbiota [151,168].

#### 3.3.2. Vitamin D/VDR Signaling Pathway Enhances the Intestinal Mucosal Barrier Integrity

A complicated intestinal barrier, consisting of the gut microbiota, mucus, epithelial cells, and immune cells, is responsible for the healthy surface of the intestinal lumen. Problems with the barrier operation or minor alterations in immune system components, microbes, mucus, or epithelial cells might contribute to many illnesses [171]. The epithelium of the gut continuously interacts with the surrounding environment. To preserve homeostasis and inhibit particular microbial species from invading or over-colonizing an area, epithelial surfaces must have enough barrier integrity and antimicrobial activity. A working mucus layer and an intact intestinal epithelium are the most important defensive mechanisms against harmful organisms. Vitamin D helps maintain this barrier function [172].

Paracellular permeability occurs through intercellular junctional complexes such as tight junctions, adherens junctions, and desmosomes. An impairment in the integrity of tight junctions leads to an increase in cellular permeability, the encountering of bacterial toxins, and the production of pro-inflammatory cytokines, which activate the immune cells and cause chronic inflammation. Vitamin D is essential for maintaining the integrity of the intestinal epithelium and modulating intestinal epithelial cell function by sustaining the expression of tight junction proteins (claudin, occludin, ZO-1, ZO-2, and vinculin), promoting the synthesis of tight junction proteins, strengthening intercellular connections, preventing cytokine-induced cell death, and protecting the gut mucosa from infections and toxins to reduce inflammation [173,174].

Mice with intestinal epithelial cells lacking VDR showed decreased levels of claudin-2 in terms of mRNA and protein. Studies examining the role of intestinal epithelial VDR and its regulatory mechanisms on claudin-2 have shown that VDR transcriptionally regulates claudin-2 expression in the intestinal epithelium of VDR∆IEC mice, which lack VDR specifically in intestinal epithelial cells under physiologically healthy conditions [175,176]. Recent research indicates that Vitamin D supplementation reduces the transmigration of *Campylobacter jejuni* by restoring the correct function of the intestinal epithelium membrane [177].

In addition to the above-mentioned studies examining the effect of vitamin D on gut microbiota composition, intestinal barrier integrity, and immune regulation, the results of several animal and human studies are summarized in Table 2 to provide a broader perspective. The connection between the immune system and microbiome is clear, with vitamin D as a crucial intermediary. Vitamin D influences immune cells to promote an anti-inflammatory environment while impacting bacterial communities, gut barrier function, epithelial integrity, and microbiota composition [172]. Improving our understanding of how vitamin D supplementation impacts bacterial communities in autoimmune diseases, including MS, is crucial.

## 4. Therapeutic Implications and Challenges

A review of studies revealed that vitamin D deficiency is seen in individuals with MS [190,191,192,193] and that there may be a correlation between MS risk and vitamin D deficiency [194,195,196]. Vitamin D supplementation in MS patients has been shown to have positive effects on quality of life, mental health [197], fatigue [118], Expanded Disability Status Scale, serum cytokines level [198], and new magnetic resonance imaging lesions [199]. In the systematic review by Langlois et al., no significant change in Expanded Disability Status Scale scores was observed as a result of vitamin D supplementation in 8 of 13 randomized controlled trials [199]. Vitamin D has been administered at different dosages and durations in studies on supplementation in MS and there are inconsistencies between the results. Therefore, it is difficult to reach a clear conclusion about the optimal dose and duration. When the guidelines are evaluated, there is no clear recommendation for supplementation [200,201]. The National Institute for Health and Care Excellence’s “Multiple sclerosis in adults: management” guideline states “Do not offer vitamin D solely for the purpose of treating MS.” [200]. In the Physicians Committee for Responsible Medicine’s Nutrition Guide for Clinicians (2023), vitamin D is referred to as “Supplementation of vitamin D and sun exposure are associated with reduced risk. Limited evidence suggests that vitamin D may play a preventive role.” [201].

Individual differences in vitamin D metabolism and requirements (genetic factors, lifestyle, and environmental factors) should be taken into account when considering supplementation. In the case of supplementation, regular monitoring of blood levels is important. It is important to emphasize safe exposure to sunlight, diet, food fortification, and supplementation as a whole [202]. In addition to increasing randomized controlled trials to determine the dose and duration of vitamin D supplementation in MS patients, it is important to conduct studies by creating formulations such as liposomal, gold, and protein nanoparticles to increase the bioavailability of supplements [203,204,205]. Studies that evaluate the effects of vitamin D on the microbiota and immune system together in MS need to be conducted. Despite this, although research has been performed to assess how vitamin D supplements affect the immune system [206,207,208,209] and microbiota [128,184,210,211] separately, there are no studies that evaluate the effect as a triplet in MS. Consequently, it is essential to clarify the precise mechanism by which vitamin D influences the immune system and microbiome in MS.

High-dose vitamin D supplementation is an important consideration. Long-term supplementation at levels exceeding 10,000 IU per day is frequently associated with toxic outcomes [212]. Studies have been carried out to assess the effects of high-dose vitamin D supplementation in people with MS [213,214,215,216]. As a result of these studies, it has been shown that high-dose vitamin D has a positive effect [213,216] or is ineffective [214,215]. It has also been stated that no negative effects have been shown at high doses [213,214,215]. In the study by Stein et al., no significant therapeutic advantage was found between high-dose (6000 IU) and low-dose vitamin D_2_ (1000 IU) supplementation in patients with RRMS [217].

In a person with MS who received high-dose vitamin D supplements for 20 months (average daily 130,000 IU and total 78,000,000 IU), hypercalcemia associated with high vitamin D concentrations caused acute kidney injury and vitamin D toxicity [218]. Nevertheless, other research indicates that high-dose vitamin D treatment has no adverse impact on MS [214]. Furthermore, it has been shown that progressive MS and symptoms of acute or chronic vitamin D intoxication (gastric symptoms, burnout, muscle weakness, etc.) overlap. Therefore, it is suggested that the diagnosis of MS may be delayed via vitamin D supplementation. Therefore, it is important to pay attention to the dosage in supplementation [119].

It highlights the importance of customized vitamin D dosing. To guarantee safety and efficacy, the determination of customized doses of vitamin D should be based on individual health profiles and starting levels of vitamin D. High-dose administration should be avoided unless there is a specifically documented deficiency and indication. Continuous monitoring of 25(OH) D_3_ levels is required to minimize the risks during high-dose administration [212].

Digital health technologies and mobile devices may be critical in monitoring vitamin D status, managing MS, and advancing personalized nutrition strategies. Wearable devices with ultraviolet sensors and mobile applications that calculate optimal sun exposure based on geographic and meteorological data can support endogenous vitamin D synthesis by providing individualized recommendations. In parallel, digital tools that track supplementation adherence and send personalized reminders contribute to preventing deficiencies, particularly in at-risk populations. These tools determine the best times and durations for sun exposure, and also suggest suitable sun protection methods based on skin type, natural sun sensitivity, and current ultraviolet exposure. These applications can track the consumption of dietary supplements, remind users to be consistent, and offer personalized suggestions based on the information above. This personalized information enables specific recommendations to be made to safely achieve adequate vitamin D levels [219,220,221]. However, most apps estimate vitamin D intake based on food databases that may lack accurate or complete data due to variations in brands, seasons, and preparation methods. They also rely on self-reported dietary intake and overlook key factors affecting sun-derived vitamin D synthesis, such as skin type, clothing, and outdoor exposure.

Furthermore, they cannot measure real-time blood vitamin D levels or fully personalize recommendations based on individual health conditions, making their estimates inherently limited [220]. For individuals with MS, mobile health platforms play an important role in personalized disease management and regular entry and monitoring of symptoms. In addition, integrating genetic data, biochemical markers, and dietary intake into digital platforms can contribute to developing personalized nutrition plans [222,223].

## 5. Conclusions

Complex interactions between the immune system, environmental factors, genetics, and the gut microbiota characterize MS as a multifactorial disease. Recent evidence suggests that dysbiosis, increased intestinal permeability, and the related immune dysregulation play important roles in pathogenesis and MS progression. Vitamin D is a critical regulator affecting both immune responses and microbial homeostasis. Vitamin D regulates gut microbiota composition via VDR, increases the expression of antimicrobial peptides, supports epithelial barrier integrity, and suppresses pro-inflammatory immune mechanisms. Vitamin D deficiency may facilitate the onset and exacerbation of MS by increasing microbial imbalance and supporting pro-inflammatory immune responses. However, gut microbiota may also affect vitamin D metabolism and signaling, suggesting a reciprocal relationship. Although the individual roles of vitamin D, gut microbiota, and immune mechanisms in MS are increasingly understood, studies addressing these factors are limited. Better-designed clinical trials and mechanistic studies are needed to reveal causal relationships and optimize treatment protocols. Elucidating the molecular mechanisms of the vitamin D–gut microbiota–immune system axis may contribute to understanding MS and other immune-related diseases and developing new treatment methods. Considering the differences in individual vitamin D metabolism due to genetic, environmental, and lifestyle factors, it is understood that personalized supplementation strategies are more effective and safer. Future research should focus on better understanding the synergistic impacts of vitamin D on the immune system and gut microbiota and investigating innovative formulations that increase bioavailability. In addition, integrating digital health technologies offers significant opportunities for monitoring, compliance, and personalization of vitamin D strategies. However, the limitations of current mobile applications highlight the need for more accurate, personalized, and biologically informed digital tools. Artificial intelligence-supported analyses can help determine which microbiota changes affect the course of MS by evaluating large data sets. Personalized medical approaches based on microbiota profiling should be emphasized, and studies should be conducted in this direction. In conclusion, personalized and evidence-based vitamin D supplementation approaches supported by technological innovations and clinical research are critical in optimizing outcomes in MS management.

## Figures and Tables

**Table 1:** The role of gut microbiota in MS.

Role of Gut Microbiota	Mechanism of Action	References
Gut microbiota alterations	Statistically significant differences in both alpha-diversity and beta-diversity Decrease in SCFA-producing bacteria such as *Firmicutes*, *Roseburia*, *Coprococcus*, *Lachnospiraceae*, *Butyricicoccus*, *Faecalibacterium*, *Dorea*, *Lachnospira*, and *Prevotella* Increase certain bacterial genera associated with pro-inflammatory responses, including *Bacteroidetes*, *Akkermansia*, and *Ruminococcus* species	[40]
Gut-associated immune system	Increased Th1 and Th17 responses due to dysbiosis (microbial antigens, microbial metabolites or dendritic cell mediated effect) Decreased Treg cells due to dysbiosis Autoimmunity due to increased Th1 and Th17 responses Increased levels of pro-inflammatory cytokines Microbial metabolites such as SCFAs alter the expression of immune genes by acting as histone deacetylase inhibitors	[75,79,85,86,89,96]
Gut–brain axis	Decreased production of SCFAs and impaired intestinal barrier integrity due to dysbiosis (leaky gut) CNS effects and neuro-inflammation due to the passage of exogenous molecules such as bacterial products and metabolites into the bloodstream due to increased intestinal permeability Impaired synthesis of neurotransmitters such as gamma-aminobutyric acid and dopamine due to dysbiosis	[75,93,94,108,109]

Abbreviations: CNS, central nervous system; Th, T helper; and SCFAs, short-chain fatty acids.

**Table 2:** The effects of vitamin D supplementation on gut microbiota, intestinal barrier integrity, and immune regulation.

Sample	Mechanism of Action	References
Effect of vitamin D supplementation on gut microbiota and intestinal barrier integrity		
Male C57BL/6 J mice	Prevention of endotoxemia and maintenance of intestinal barrier function Serum LPS concentration ↓ Caecum ZO-1 and Occludin mRNA relative expression ↑ Improved α-diversity and β-diversity of the gut microbiota Relative abundance of *Firmicutes* ↓ Relative abundance of *Bacteroidetes* and *Proteobacteria* ↑ Gut dysbiosis ↓	[178]
Non-obese diabetic mice	Abundance of *Lachnospiraceae_FCS020* and *Ruminiclostridium_9 ↑* Abundance of *Marvinbryantia* ↓	[179]
C57BL/6J Mice	Colonic ZO-1 and Occludin mRNA relative expression ↑ Relative abundance of *Dubosiella newyorkensis* ↑	[180]
Female C57BL/6J mice	Plasma TMA and TMAO ↓ Increased gut microbial α-diversity indices Relative abundance of *Firmicutes* ↓ Relative abundance of *Bacteroidetes* ↑ *Bacteroidetes/Firmicutes* ratio ↑ Relative abundance of *Akkermansia* and *Ruminiclostridium* ↑	[181]
Male BALB/C mice	Percentages of *Pseudomonas aeruginosa* and *Salmonella/Shigella* spp. ↓	[182]
Healthy adults	*Bifidobacteriaceae* family ↑	[183]
	Increased gut microbial diversity Relative abundance of *Firmicutes* ↓ Relative abundance of *Bacteroidetes* ↑ *Bacteroidetes/Firmicutes* ratio ↑ Relative abundance of *Actinobacteria* and *Verrucomicrobia* ↑ Relative abundance of *Bifidobacterium* and *Akkermansia ↑* Relative abundance of *Roseburia*, *Ruminococcus*, and *Fecalibacterium* ↓	[128]
	Relative abundance of *Faecalibacterium* spp., *Clostridia*, and *Ruminococcaceae ↓* *Firmicutes/Bacteroidetes* ratio ↓	[130]
	No difference was observed in terms of α-diversity. Abundance of genus *Lachnospira ↑* Abundance of genus *Blautia* ↓ Abundance of genus *Coprococcus ↑* (>75 nmol/L) Abundance of genus *Ruminococcus* ↓ (<50 nmol/L)	[184]
MS	Majority of operational taxonomic units ↓ *Akkermansia*, *Faecalibacterium*, and *Coprococcus* in untreated MS *↑*	[132]
Older adults (60–84 y)	No significant difference was observed in gut microbiota composition	[185]
Infants	Abundance of genus *Bilophila*, *Megamonas* and *Peptostreptococcus* ↓	[186]
	Relative abundance of *Bifidobacterium*, *Streptococcus*, and *Lactobacillus ↑* Proportion of Bifidobacterium correlated vitamin D circulating level	[187]
*Clostridioides Difficile* Infected Patients	Abundance of genus *Proteobacteria*, *Enterobacteriaceae* and *Escherichia* ↓ Abundance of *Lachnospiraceae*, *Ruminococcaceae*, *Christensenellaceae*, *Bifidobacteriaceae*, and *Sutterellaceae ↑*	[188]
Effect of vitamin D supplementation on immune regulation		
Male C57BL/6 J mice	TNF-α, IL-1β ↓	[178]
Non-obese diabetic mice	Splenic FoxP3^+^ Treg Cells ↑ IL-10 secretion CD4^+^ T Cells ↑ Type 1 Diabetes Mellitus incidence ↓	[179]
Weaned C57BL/6 mice	Inhibit pro-inflammatory cytokines including IL-1, IL-8, IL-17, and TNF-α	[189]
25-hydroxyvitamin D-deficient RRMS patients	↓ IFN-γ secretion by CD4^+^ T cells received 10,000 IU/week cholecalciferol for 3 months	[156]
RRMS patients	High-dose group (received 10,000 IU/week cholecalciferol for 6 months) ↓ IL-17 production by CD4^+^ T cells	[157]
RRMS patients	Control group (received 50,000 IU cholecalciferol every 5 days for 12 weeks) ↓ IL-17 levels	[158]
MS patients	Received 50,000 IU cholecalciferol once weekly ↓ TNF-α:IL-5 ratio, ↓ TNF-α:IL-6 ratio, ↓ TNF-α:IL-10 ratio, and ↓ IFN-γ:IL-10 ratio at the 12-month follow-up	[159]
RRMS patients	Received 20,000 IU/d cholecalciferol for 12 weeks ↑ %IL-17 production by CD4^+^ T cells	[160]

Abbreviation: TMA, trimethylamine; TMAO, trimethylamine-n-oxide; FoxP3^+^, forkhead box P3; IFN-γ, interferon gamma; IL, interleukin; MS, multiple sclerosis; LPS, lipopolysaccharides; RRMS, relapse–remitting multiple sclerosis; TNF-α, tumor necrosis factor alpha; ZO-1, zonula occluden-1; mRNA, messenger ribonucleic acid; ↑, increase; and ↓, decrease.

## Data Availability

No new data were created or analyzed in this study. Data sharing is not applicable to this article.

## Abbreviations

The following abbreviations are used in this manuscript:

MS	Multiple Sclerosis
VDR	Vitamin D receptor
Th	T Helper Cells
CNS	Central Nervous System
RRMS	Relapsing–Remitting Multiple Sclerosis
PPMS	Primary Progressive Multiple Sclerosis
SPMS	Secondary Progressive Multiple Sclerosis
SCFAs	Short-Chain Fatty Acids
Tregs	T Regulatory Cells
IL	Interleukin
TNF-α	Tumor Necrosis Factor Alpha
AhR	Aryl Hydrocarbon Receptor
FGF-23	Fibroblast Growth Factor-23
RXR	Retinoid-X Receptor
AMPs	Antimicrobial Peptides
ZO	Zonula Occludens
